# Effects of Blood Flow Restriction on O_2_ Muscle Extraction and O_2_ Pulmonary Uptake Kinetics During Heavy Exercise

**DOI:** 10.3389/fphys.2021.722848

**Published:** 2021-09-01

**Authors:** Killian Salzmann, Anthony M. J. Sanchez, Fabio Borrani

**Affiliations:** ^1^Institute of Sport Sciences of University of Lausanne (ISSUL), University of Lausanne, Lausanne, Switzerland; ^2^University of Perpignan Via Domitia (UPVD), Faculty of Sports Sciences, Laboratoire Interdisciplinaire Performance Santé Environnement De Montagne (LIPSEM), Font-Romeu, France

**Keywords:** blood flow restriction, slow component of oxygen consumption, cycling exercise, oxygen extraction, skeletal muscle, vascular occlusion, NIRS, tissue saturation index

## Abstract

This study aimed to determine the effects of three levels of blood flow restriction (BFR) on $\dot{V}O_{2}$ and *O*_2_ extraction kinetics during heavy cycling exercise transitions. Twelve healthy trained males completed two bouts of 10 min heavy intensity exercise without BFR (CON), with 40% or 50% BFR (BFR40 and BFR50, respectively). $\dot{V}O_{2}$ and tissue saturation index (TSI) were continuously measured and modelled using multiexponential functions. The time constant of the $\dot{V}O_{2}$ primary phase was significantly slowed in BFR40 (26.4 ± 2.0s; *p* < 0.001) and BFR50 (27.1 ± 2.1s; *p* = 0.001) compared to CON (19.0 ± 1.1s). The amplitude of the $\dot{V}O_{2}$ slow component was significantly increased (*p* < 0.001) with BFR in a pressure-dependent manner 3.6 ± 0.7, 6.7 ± 0.9 and 9.7 ± 1.0 ml·min^−1^·kg^−1^ for CON, BFR40, and BFR50, respectively. While no acceleration of the primary component of the TSI kinetics was observed, there was an increase (p < 0.001) of the phase 3 amplitude with BFR (CON −0.8 ± 0.3% VS BFR40 −2.9 ± 0.9%, CON VS BFR50 −2.8 ± 0.8%). It may be speculated that BFR applied during cycling exercise in the heavy intensity domain shifted the working muscles to an *O*_2_ dependent situation. The acceleration of the extraction kinetics could have reached a plateau, hence not permitting compensation for the slowdown of the blood flow kinetics, and slowing $\dot{V}O_{2}$ kinetics.

## Introduction

Aerobic training with blood flow restriction (BFR) was found to be beneficial for enhancing adaptations, including endurance and muscle hypertrophy (Pignanelli et al., 2021; Preobrazenski et al., 2021). These results are thought to be caused by some acute effects of BFR reducing venous blood flow according to the level of pressure exerted. Thus, BFR may generate local hypoxia and an accumulation of acidity and metabolites in the working muscles. BFR application has been suggested to increase neuromuscular activation of type II fibres (Moritani et al., 1992) and enhance of cardiac and ventilatory responses due to stimulation of group III and IV afferents (Adreani and Kaufman, 1998). These effects have been reported to cause an elevation of the total energy expenditure resulting in a loss of efficiency observed by an increase of the pulmonary oxygen uptake ($\dot{V}O_{2}$) during submaximal exercise (Mendonca et al., 2014; Silva et al., 2019). However, most of the studies about the acute effects of BFR on response to aerobic exercise used steady state measurements that could not accurately represent the dynamic regulation of the metabolism.

For decades, it has been observed that during a transition from rest to moderate exercise, $\dot{V}O_{2}$ increases in an exponential fashion to reach a steady state resulting in *O*_2_ deficit in the early phase of exercise (Hill and Lupton, 1923). This phenomenon known as “oxidative inertia” comprises a cardiodynamic and a primary phase that can be evaluated by the time constant of the primary component ($\dot{V}O_{2}\tau p$) of $\dot{V}O_{2}$ response (Poole and Jones, 2012). This parameter provides insight into the energetic metabolism dynamics and is suggested to be related to aerobic health (Bauer et al., 1999) and performance (Burnley and Jones, 2007). About 3 min following the start of an exercise conducted in the heavy-intensity domain above the first ventilatory threshold (VT1), the primary component is superimposed by an elevation of $\dot{V}O_{2}$ known as the $\dot{V}O_{2}$ slow component (SC). $\dot{V}O_{2}$ SC further delays the stabilisation of the oxidative response and is thought to be related to a deterioration of muscular efficiency. Thus, an increase of the ATP required for a similar power output is observed concomitantly to increases of the ventilatory, cardiac and auxiliary muscles work (Henson et al., 1989; Krustrup et al., 2004; Jones et al., 2011; Korzeniewski and Zoladz, 2015). Accordingly, a theoretical study has recently suggested that changes in $\dot{V}O_{2}$ kinetics may be related to OXPHOS (mitochondrial oxidative phosphorylation system) activity and to additional ATP usage that appears when inorganic phosphate (Pi) exceeds a critical value called Pi_crit_ (Allen and Westerblad, 2001; Korzeniewski and Rossiter, 2021). However, recent works have also suggested that $\dot{V}O_{2}$ SC was mainly due to a metabolic shift corresponding to enhanced $\dot{V}O_{2}$, thus compensating a reduction of the anaerobic energy contribution without changes of total energy utilisation during heavy intensity exercises (Colosio et al., 2020). Thus, the occurrence of $\dot{V}O_{2}$ SC may be explained by a shift between metabolic energy sources in heavy and a loss of muscle efficiency in severe intensity domain (Conde Alonso et al., 2020; Pignanelli et al., 2021; Preobrazenski et al., 2021).

Modelling $\dot{V}O_{2}$ kinetics provides additional information about the different mechanisms affecting the dynamic regulation of the oxidative metabolism during exercise. However, to the best of our knowledge, there is few studies that aimed to analyze the effects of BFR on response to aerobic exercise through this approach (Knight et al., 2004). Nonetheless, a study applying BFR during cycling exercise in the moderate intensity domain found an increase of $\dot{V}O_{2}$ at the end of exercise due to the appearance of a $\dot{V}O_{2}$ SC. This suggests that BFR increased the metabolic cost of exercise (Knight et al., 2004). Although this observation is in concert with studies that observed an increase of the metabolic activity, $\dot{V}O_{2}$ SC only occurred at the end of the exercise. Moreover, $\dot{V}O_{2}$ still tended to increase during the last 30 s suggesting that stabilisation wasn't fully reached. Therefore, it seems that exercise bouts of longer duration are needed to completely analyze the $\dot{V}O_{2}$ kinetics during BFR. The authors suggested that BFR enhanced mechanisms involved in occurrence of $\dot{V}O_{2}$ SC, including Pi accumulation, enhanced ventilation and neuromuscular recruitment (Knight et al., 2004). Increased amplitude of $\dot{V}O_{2}$ SC has also been reported during ischemia induced by supine cycling in the heavy-intensity domain (Koga et al., 1999). Interestingly, some studies also using supine cycling found a deceleration of the primary phase suggested to be caused by the reduction of the *O*_2_ delivery in the supine position (Hughson et al., 1991; MacDonald et al., 1998). Thus, it seems that ischemia can alter the $\dot{V}O_{2}$ kinetics by slowing the primary phase and by increasing the amplitude of $\dot{V}O_{2}$ SC.

However, little is known about the impact of BFR on $\dot{V}O_{2}$ kinetics, especially during high-intensity exercises. In addition to ischemia, the possible reduced venous return (as suggested by diminution of stroke volume) may alter the clearance of muscle metabolites and acidity (Scott et al., 2015). There is also evidence of reduced muscle (or limb) blood flow “during” exercise with an applied BFR (Willis et al., 2019). Tissue saturation index (TSI), measured by near infrared spectrometry (NIRS), allows the estimation of local muscular *O*_2_ extraction, which is used as a proxy of arteriovenous difference and can give insight into the dynamic equilibrium between *O*_2_ delivery and utilisation (Grassi and Quaresima, 2016). *In situation* of slowed kinetics of *O*_2_ delivery, such as during intense exercise transitions, it has been suggested that *O*_2_ extraction increases more rapidly to maintain the acceleration of $\dot{V}O_{2}$ (Jones et al., 2012). It has been shown that the level of occlusion decreased blood flow and increased TSI during arm exercise but, the magnitude of these effects isn't linear (Kilgas et al., 2019).

Therefore, the goal of this study was to determine the effects of different levels of BFR on $\dot{V}O_{2}$ kinetics during cycling exercise transitions performed in the heavy intensity domain. The main hypothesis is that BFR would slow the primary component and increase $\dot{V}O_{2}$ SC amplitude of the $\dot{V}O_{2}$ kinetics as a function of the pressure applied. Moreover, BFR application would cause an acceleration of *O*_2_ extraction to compensate blood flow alteration.

## Methods

### Subjects

Twelve healthy trained (mean ± SD, age 24.3 ± 2.7 years; weight 74.9 ± 8 kg; height 181 ± 6 cm and body fat percentage 11.1 ± 1.8%; peak oxygen consumption, $\dot{V}O_{2}$peak 62.5 ± 7.4 ml·min^−1^·kg^−1^) male participants took part in this experiment. Prior to the first visit, the athletes were informed about the experimental procedures and the possible risks and discomforts. The participants provided written informed consent and completed a questionnaire to exclude all potential cardiorespiratory and injury risks. The experimental protocol was approved by the “Commission cantonale d'éthique de la recherche sur l'être humain, Canton de Vaud, CER-VD” (VD-2017-02193). All experiments were performed in accordance with relevant guidelines and regulations.

### Experimental Design

The experimental protocol consisted of four sessions over 4 weeks. The sessions lasted between 60 and 90 min and were separated by at least 48 h to avoid fatigue related to the experimentation. During the first session (familiarisation session) anthropometric, total femoral artery occlusion pressure measurements and a ramp cycle ergometer test were carried out. The three following sessions (kinetics sessions) were the experimental phases. Here, the purpose was to determine $\dot{V}O_{2}$ kinetics during three distinct conditions: without blood flow restriction (CON), and with 40% (BFR40) and 50% (BFR50) occlusion of the total femoral artery pressure. A total of six measurements including two measurements per experimental condition distributed randomly were taken.

### Familiarisation Session

Anthropometric measurements were performed before the ramp test for height, weight, and body composition determination. Body composition was estimated using the four skinfold thickness method (Durnin and Womersley, 1974). The participants sat on a chair for the measurement of the total femoral artery occlusion pressure. The cuffs (*SC10D, cuff size 11x85 cm, bladder size 10x41 cm*) were placed around the right inferior limb proximal to the hip articulation. The occlusion pressure was then progressively increased with the inflation apparatus (*E20/AG101 Rapid Cuff Inflation System, D.E Hokanson Inc., Bellevue, WA, United-States*). The occlusion level was determined with an ultrasound probe (*L 12-5L60N, ClarUs EXT, Telemed Medical Systems, Milan, Italy*) to measure blood flow. Total occlusion pressure was reached when arterial blood flow was no detectable. At least two measurements were performed and averaged to obtain the most representative value. When there was a difference of more than 5% between the two measurements, a third evaluation was completed. The value of total occlusion pressure was then used to determine the different pressures applied for the BFR conditions during the exercise sessions. The participants performed the ramp test on a cycle ergometer (*Lode Excalibur Sport, Groningen, The Netherlands*). The protocol began with a three min rest followed by a five min warmup at 60 W. Directly after warmup, the ramp phase started at an intensity of 60 W with a 1 W increment every 2 s. The athletes had to maintain a stable pedal rate between 70 and 100 rpm throughout the test until volitional termination. Breath-by-breath gas exchanges were measured continuously during the test with a gas exchange analyzer (*COSMED QUARK, Italy*). The gas exchange analyzer was calibrated according to the manufacturer's instructions using a 3-l syringe (*Hans Rudolph Inc, Shawnee, Kansas, United-States*) for volume and ambient air and known gas mix (*O*_2_ = 15%, *CO*_2_ = 5%) for O_2_ and CO_2_ analyzer. VT1 and $\dot{V}O_{2}$peak were determined to define the power equivalent at 10% (PΔ10) of the difference between the power at VT1 and the maximal aerobic power.

### Exercise Sessions

The three exercise sessions were composed of two exercises separated by a 45 min rest to avoid the alteration of the $\dot{V}O_{2}$ kinetics and to restore baseline metabolic parameters (Burnley et al., 2006). Exercise was composed of 3 min of rest followed by 3 min of empty pedalling and 10 min at PΔ10 in a random experimental condition. During BFR conditions, the cuffs were applied on both inferior limbs proximal to the hip articulation and inflated at the beginning of the 10 min at PΔ10. The pedal rate had to be stable and similar between each measurement due to a possible effect of this parameter on the $\dot{V}O_{2}$ kinetics (Pringle et al., 2003). Gas exchanges and muscular *O*_2_ extraction were measured throughout the exercise. Muscular *O*_2_ extraction measurements were monitored by an NIRS probe (Portalite, Artemis, The Netherlands) and placed on the distal portion of the right vastus lateralis muscle. The device was maintained positioned by an elastic band wrapped around it to minimise the possibility of extraneous light. NIRS device includes three transmitters situated at 3.5, 4, and 4.5 cm from the receptor and the acquisition frequency was 50 Hz. Two different wavelength laser diodes provided the light source (760 and 850 nm), and the differential pathlength factor was set to four.

### Data Analysis

During the progressive ramp test, $\dot{V}O_{2}$ measurements were averaged at 10 s intervals. VT1 was determined according to three criteria: (i) excess of carbon dioxide production ($\dot{V}CO_{2}$) compared to $\dot{V}O_{2}$, represented by an inflexion point on the $\dot{V}O_{2}-\dot{V}CO_{2}$ relation curve (Beaver et al., 1986); (ii) hyperventilation relative to $\dot{V}O_{2}$, identified by a systematic increase of ventilation ($\dot{V}E$) in relation to $\dot{V}O_{2}$; (iii) the exclusion of hyperventilation relative to $\dot{V}CO_{2}$ corresponding to a continuous decreasing or stable state of the *CO*_2_ respiratory equivalent ($\dot{V}E$/$\dot{V}CO_{2}$) during the events of (i) and (ii) (Levett et al., 2018). $\dot{V}O_{2}$peak was calculated as the highest 30 s $\dot{V}O_{2}$ average measured during the ramp test (Myers et al., 1990). The maximal aerobic power was determined as the lowest intensity eliciting a plateau in the $\dot{V}O_{2}$-power relationship. The $\dot{V}O_{2}$ data were modelled and analysed with a computer software (*Microsoft Excel, Microsoft Corporation, Redmond, WA, United-States*). Raw data were first cleared of artefacts through the deletion of values with a difference of more than three SD from the local average (Lamarra et al., 1987). Then, both measurements from each subject at the same condition were second by second linearly interpolated, time aligned, averaged. Because the number of participants was small (12 subjects) and consequently the distribution of data from the reference samples was non-Gaussian, and because it provides reliable and more powerful results in many conditions, and it does not require any assumptions as opposed to other alternatives, bootstrapping method was used (Dwivedi et al., 2017). The bootstrap sample was the same size as the original dataset (12) and was built using sampling with replacement. This process was repeated 1,000 times, and for each of these bootstrap samples, the model parameters were computed for each condition as follow:

(1) $$\begin{array}{l} \dot{V}O_{2}\left(t\right)\;=\;\dot{V}O_{2BL}+Ap\left(1-e^{-\left(t-TDp\right)/\tau p}\right)\\ \;+Asc\;\left(1-e^{-\left(t-TDsc\right)/\tau sc}\right) \end{array}$$

where $\dot{V}O_{2}\left(t\right)$ represents $\dot{V}O_{2}$ as a function of time; $\dot{V}O_{2BL}$ is the $\dot{V}O_{2}$ averaged during the last 30 s of the empty pedalling phase; *Ap*, *TDp*, and τ*p* are the amplitude, the time at the beginning and the time constant respectively of the primary phase; *Asc*, *TDsc*, and τ*sc* are the amplitude, the time at the beginning and the time constant respectively of $\dot{V}O_{2}$ SC. The mean response time (*MRT*) was calculated as *TDp* + τ*p*. Because suggested of not reflecting the $\dot{V}O_{2}$ from the working muscles, the cardiodynamic phase was not considered. Therefore, the first 20 s of exercise were not modelled. $\dot{V}O_{2}$ SC amplitude (*Asc*′) at the end of exercise was calculated as follow:

(2) $$\begin{array}{l} Asc^{\prime }=Asc\;\left(1-e^{-\left(tend-TDsc\right)/\tau sc}\right) \end{array}$$

where *tend* corresponds to exercise duration. $\dot{V}O_{2}end$ is the last value of modelled $\dot{V}O_{2}$. The total amplitude ($\dot{V}O_{2}Atot$) is obtained by the $\dot{V}O_{2}end\;-\;\dot{V}O_{2BL}$ calculation.

**Figure 1:**
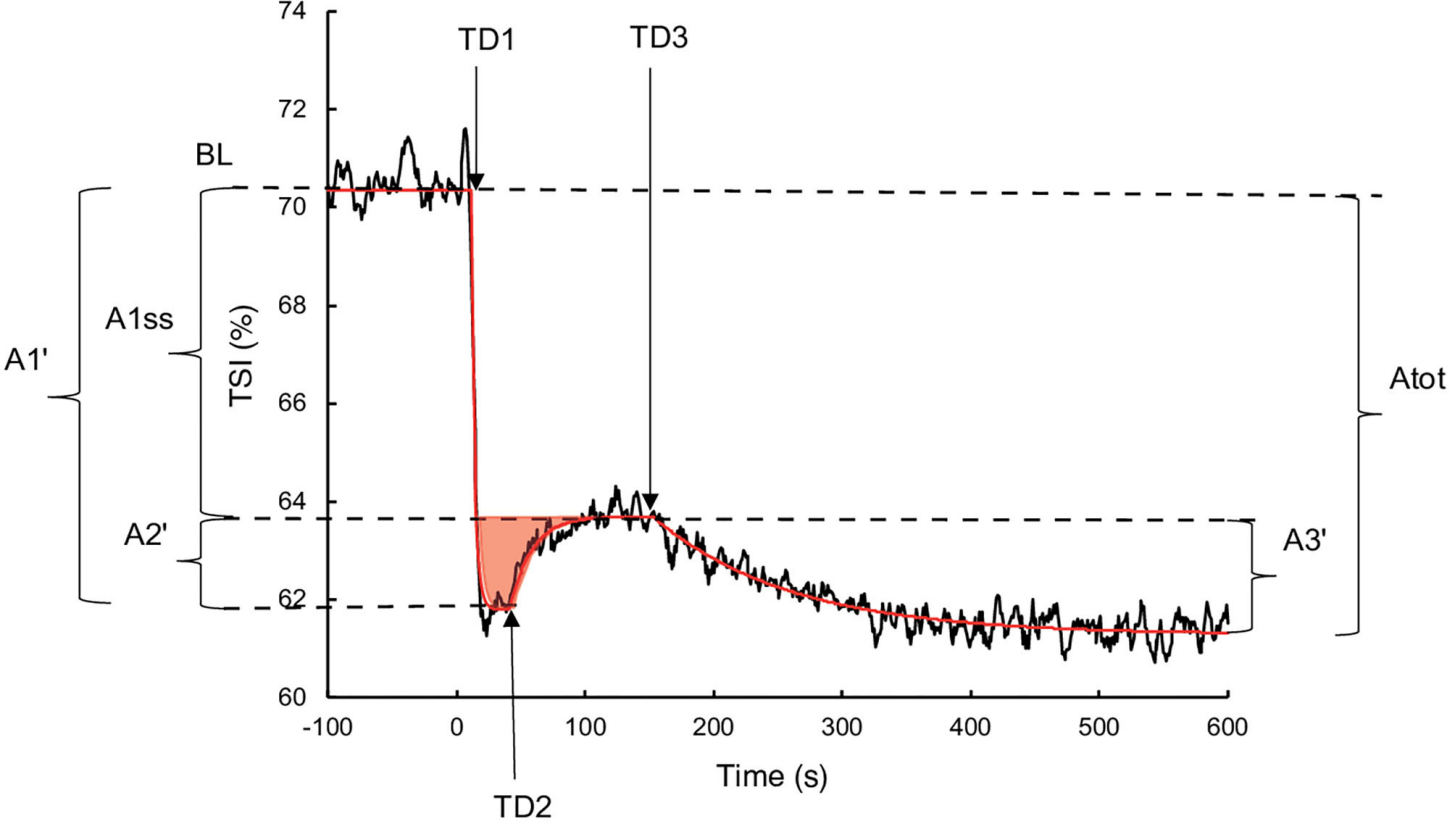
Illustration of the three components of TSI kinetics for a representative subject during exercise without occlusion. The black line represents the TSI measurement, and the red line corresponds to TSI modelling. The red surface represents the TSI overshoot. BL: Baseline; *A*1′, *A*2′, *A*1*ss*′, A3', and A*tot*: amplitudes of phase 1, phase 2, steady state, phase 3, and total response, respectively; *TD*1, *TD*2, and TD3: time delays of phase 1, phase 2, and phase 3, respectively.

Oxygen extraction was estimated by the TSI from the NIRS measurement in haemoglobin (Hb) concentrations differentiating deoxyhaemoglobin (HHb) and oxyhaemoglobin (*O*_2_Hb). The TSI equalled (*O*_2_Hb/(*O*_2_Hb+HHb))x100. The exported data contained one value per second and were processed using the same procedure as the $\dot{V}O_{2}$ measurements. A Study measuring the tissue oxygenation index during transitions observed the presence of an “overshoot,” illustrated by a primary increase followed by a decrease and a stabilisation (Bowen et al., 2011). Therefore, to consider this phenomenon, the following three components exponential equation was used (Figure 1):

(3) $$\begin{array}{l} TSI\left(t\right)\;=TSI_{BL}+A1\left(1-e^{-\left(t-TD1\right)/\tau 1}\right)+A2\;\left(1-e^{-\left(t-TD2\right)/\tau 2}\right)\\ \;+\;A3\left(1-e^{-\left(t-TD3\right)/\tau 3}\right) \end{array}$$

where *TSI*(*t*) represents the TSI as a function of time; *TSI*_*BL*_ is the TSI averaged during the last 30 s of empty pedalling; *A*1, *TD*1, and τ1 are the amplitude, the time at the beginning and the time constant of the phase 1 respectively; *A*2, *TD*2, and τ2 are the amplitude, the time at the beginning and the time constant of the phase 2 respectively; *A*3, *TD*3, and τ3 are the amplitude, the time at the beginning and the time constant of the phase 3 respectively. The mean response time (*MRT*) was calculated as *TD*1+ τ1. The real amplitudes of the phases 1 (*A*1′) and 2 (*A*2′) were calculated using Equation (2) due to their truncated character. Due to the same reasons as for $\dot{V}O_{2}$, the real phase 3 amplitude (*A*3′) was also calculated using the Equation (2). The amplitude at the steady state following the completion of phase 2 (*A*1*ss*′) was calculated as *A*1′ + *A*2′. The different parameters of the TSI and $\dot{V}O_{2}$ kinetics were determined to minimise the least square sums between the model and the measurements.

### Statistical Analysis

For the comparison of $\dot{V}O_{2}$ and NIRS model parameters in different conditions, the difference between conditions for each parameter of the 1000 bootstrap samples were plotted (Curran-Everett, 2009; Millet and Borrani, 2009). From the resulting Gauss curve, if the percentile of zero difference was out of the range from the 2.5th and 97.5th percentiles, significant differences were determined. When the percentile of the zero difference was smaller than 50%, the *p*-value was then defined as the double of the percentile of zero difference, and the complement to 100% multiplied by two for values larger than 50%. The results are presented as means ± standard deviations (SDs). The *p*-value was set at 0.05.

## Results

### Effects of Blood Flow Restriction on $\dot{\text{V}}{\text{O}}_{2}$ Kinetics

**Figure 2:**
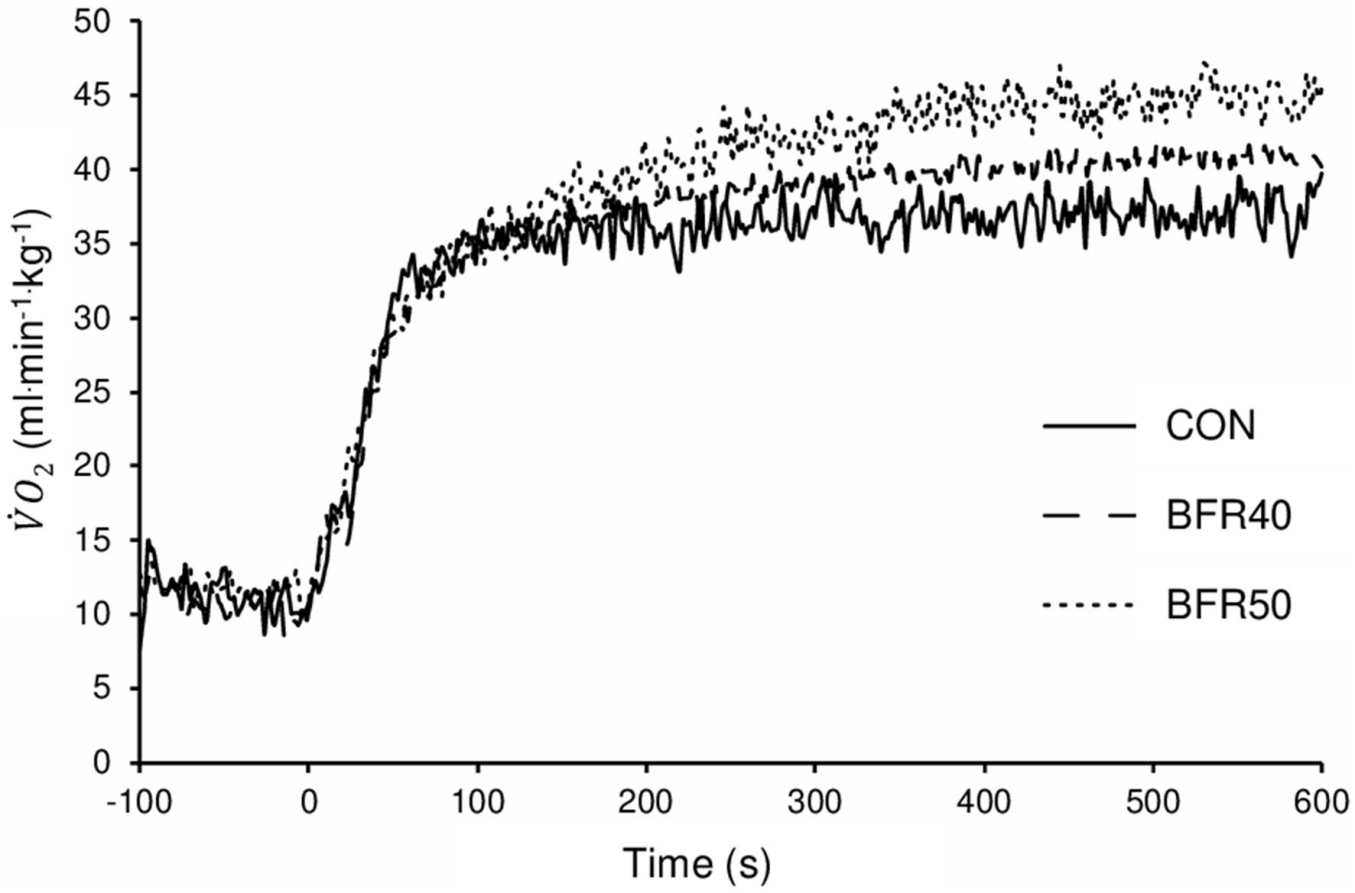
Oxygen uptake for exercise without blood flow restriction (CON), with 40% (BFR40) or 50% (BFR50) occlusion for a representative subject.

The mean BFR pressure was 76.6 ± 4.7 mmHg and 95.9 ± 5.8 mmHg during BFR40 and BFR50, respectively. $\dot{V}O_{2}$ kinetics parameters are presented in Table 1. Regarding the primary phase, $\dot{V}O_{2}Ap$ was similar between the conditions (*p* > 0.05). $\dot{V}O_{2}TDp$ was significantly lower for BFR40 compared to CON (*p* = 0.013). No significant difference was found between CON and BFR50 (*p* = 0.076) or between BFR40 and BFR50 (*p* = 0.553). Concerning $\dot{V}O_{2}\tau p$, there was an increase with BFR40 (*p* < 0.001) and BFR50 (*p* = 0.001) compared to CON but no difference was observed between BFR40 and BFR50 (*p* = 0.745). $\dot{V}O_{2}MRT$ was significantly higher in BFR40 (*p* < 0.001) and BFR50 (*p* = 0.003) compared to CON, indicating a slowdown in the early process of the $\dot{V}O_{2}$ kinetics. However, no difference was found between BFR40 and BFR50 (*p* = 0.886). Concerning $\dot{V}O_{2}$ SC, the $\dot{V}O_{2}Asc^{\prime }$ was increased with both BFR condition (*p* < 0.001) and $\dot{V}O_{2}Asc^{\prime }$ was significantly greater for BFR50 compared to BFR40 (*p* < 0.001). There were no differences of $\dot{V}O_{2}TDsc$ between conditions (*p* > 0.05). Finally, $\dot{V}O_{2}Atot$ was increased for each BFR conditions (*p* < 0.001). $\dot{V}O_{2}Atot$ was also higher in BFR50 compared to BFR40 (*p* < 0.001). The $\dot{V}O_{2}$ uptake kinetics during exercises are presented in Figure 2 for a representative participant.

**Table 1:** Oxygen uptake kinetics parameters for exercise without blood flow restriction (CON), with 40% (BFR40) or 50% (BFR50) occlusion.

	**CON**	**BFR40**	**BFR50**
$\dot{\text{V}}{\text{O}}_{2}\text{A}\text{p}$ (ml·min^−1^·kg^−1^)	26.7 ± 1.1	25.9 ± 1.2	25.8 ± 1.2
$\dot{\text{V}}{\text{O}}_{2}\text{TDp}$ (s)	16.4 ± 0.5	14.9 ± 0.7^*^	14.4 ± 1.2
$\dot{\text{V}}{\text{O}}_{2}\tau p$ (s)	19.0 ± 1.1	26.4 ± 2.0^*^	27.1 ± 2.1^*^
$\dot{\text{V}}{\text{O}}_{2}\text{MRT}$ (s)	35.4 ± 1.0	41.3 ± 1.6^*^	41.4 ± 1.6^*^
$\dot{\text{V}}{\text{O}}_{2}\text{TDs}\text{c}$ (s)	92.0 ± 8.4	100.8 ± 8.3	98.8 ± 8.6
$\dot{\text{V}}{\text{O}}_{2}\text{A}\text{s}{\text{c}}^{\prime }$ (ml·min^−1^·kg^−1^)	3.6 ± 0.7	6.7 ± 0.9^*^	9.7 ± 1.0^*^^§^
$\dot{\text{V}}{\text{O}}_{2}\text{Atot}$ (ml·min^−1^·kg^−1^)	30.3 ± 1.3	32.6 ± 1.2^*^	35.5 ± 1.7^*^^§^

*Values are means ± SDs. Ap, Asc′, and Atot, amplitudes of primary phase, slow component, and total response, respectively; TDp and TDsc, time delays of primary phase and slow component, respectively; τp, time constant of primary phase; MRT, mean response time of the primary phase*.

* *Significantly different from CON (p < 0.05)*.

§ *Significantly different from BFR40 (p < 0.05)*.

### Effects of Vascular Occlusion on the Muscular **O**_**2**_ Extraction Kinetics

**Figure 3:**
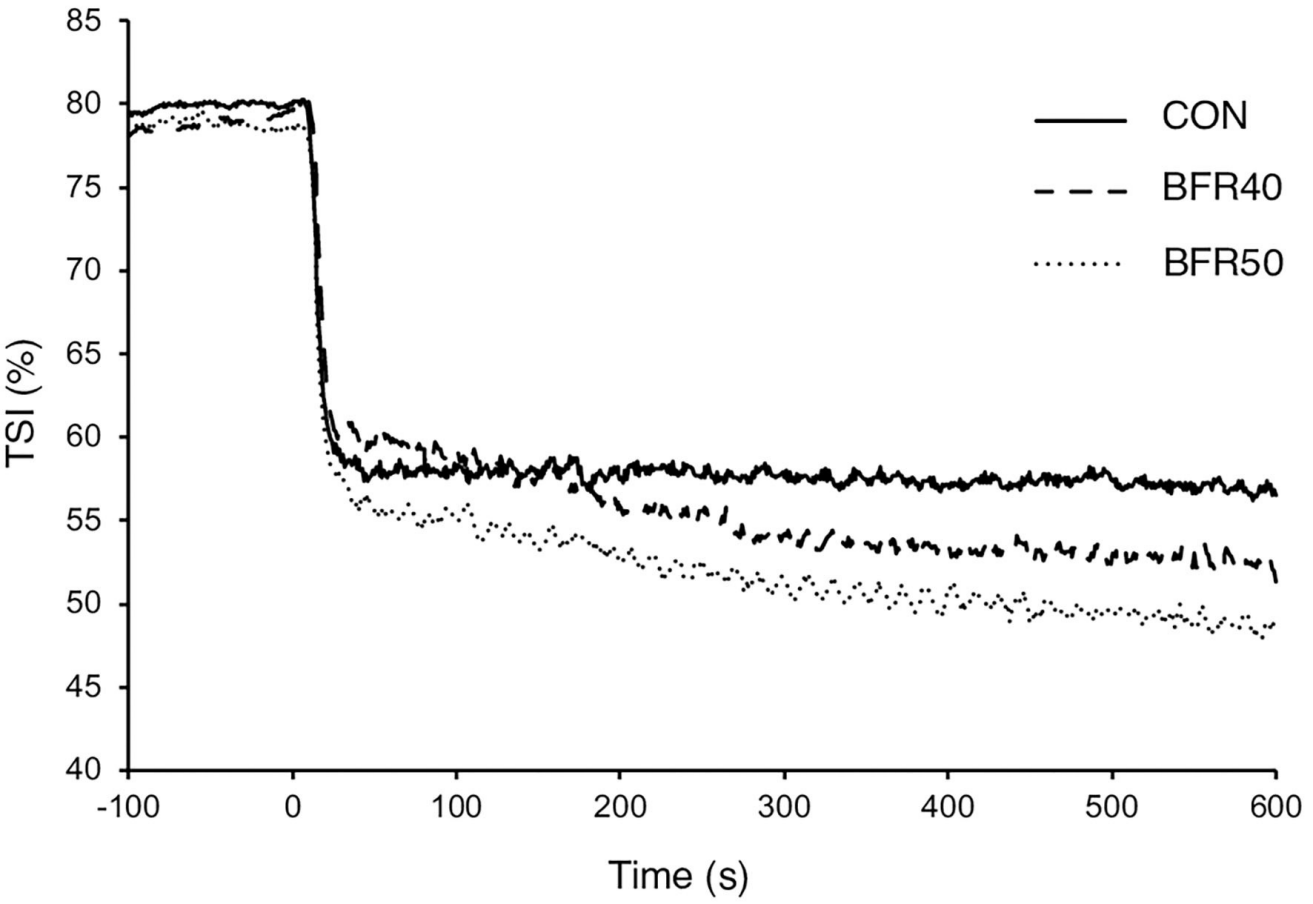
Tissue saturation index for exercise without blood flow restriction (CON), with 40% (BFR40) or 50% (BFR50) occlusion for a representative subject.

The main TSI kinetics model parameters are presented in Table 2. For the first two phases, there were no significant differences (*p* > 0.05) between conditions concerning the amplitude parameters (*TSIA*1′*, TSIA*2′, and *TSIA*1*ss*′). *TSIMRT* decreased with BFR40 (*p* = 0.041) and BFR50 (*p* = 0.005), indicating an acceleration of the early process of the extraction kinetics. Concerning phase 3, *TSIA3'* was higher in BFR40 and BFR50 than in CON (*p* < 0.001) but no difference was found between BFR40 and BFR50 (*p* > 0.05). *TSITD3* decreased with BFR40 (*p* = 0.002) and BFR50 (*p* = 0.007) but no difference was observed between BFR40 and BFR50 (*p* > 0.05). Finally, *TSIAtot* increased with BFR40 (*p* = 0.006) and BFR50 (*p* = 0.002) without any difference between BFR conditions (*p* > 0.05). Responses of muscle *TSI* during exercises are presented in Figure 3 for a representative participant.

**Table 2:** Oxygen extraction kinetics parameters for exercise without blood flow restriction (CON), with 40% (BFR40) or 50% (BFR50) occlusion.

	**CON**	**BFR40**	**BFR50**
**TSIA1′** (%)	−14.5 ± 1.1	−14.7 ± 1.2	−15.4 ± 1.6
**TSITD1** (s)	10.1 ± 0.4	8.9 ± 0.6^*^	8.9 ± 0.7^*^
**TSIτ*1*** (s)	5.7 ± 0.5	5.6 ± 0.4	5.4 ± 0.4
**TSIMRT** (s)	15.8 ± 0.4	14.5 ± 0.6^*^	14.3 ± 0.7^*^
**TSIA2′** (%)	0.8 ± 0.3	0.8 ± 0.6	0.9 ± 0.5
**TSITD2** (s)	49.6 ± 3.5	51.1 ± 11.2	47.2 ± 5.4
**TSIA1ss′** (%)	−13.7 ± 1.1	−13.9 ± 1.5	−14.6 ± 1.8
**TSITD3** (s)	190.0 ± 32.6	116.5 ± 12.6^*^	123.6 ± 19.0^*^
**TSIA3′** (%)	−0.8 ± 0.3	−2.9 ± 0.9^*^	−2.8 ± 0.8^*^
**TSIAtot** (%)	−14.6 ± 1.3	−16.8 ± 2.0^*^	−17.3 ± 2.1^*^

*Values are means ± SD. A1′, A2′, A1ss′, A3′, and Atot, amplitudes of phase 1, phase 2, steady state, phase 3, and total response, respectively; TD1, TD2, and TD3, time delays of phase 1, phase 2, and phase 3, respectively; TSI, tissue saturation index; τ1, time constant of phase 1. MRT, mean response time of phase 1*.

* *Significantly different from CON (p < 0.05)*.

## Discussion

The main purpose of this study was to examine the effects of different levels of BFR on $\dot{V}O_{2}$ kinetics during cycling exercise transitions in the heavy intensity domain. Results showed that $\dot{V}O_{2}\tau p$ was increased with BFR, suggesting a slowdown in the oxidative metabolism potentially related with the reduction of blood flow. An increase of $\dot{V}O_{2}Asc^{\prime }$ as a function of BFR pressure was also observed, indicating a greater metabolic demand. Finally, BFR did not elicit an acceleration of the primary phase of the TSI kinetics as a function of the pressure applied.

The first hypothesis proposing a slowdown in the primary phase as a function of the BFR pressure has been partially confirmed. Indeed, there was an increased $\dot{V}O_{2}\tau p$ with BFR, but no significant differences were found between the two pressures applied. This result can be explained by the fact that the reduction of blood flow is not linearly affected by the relative pressure of BFR (Kilgas et al., 2019). Alternatively, pressures of the two BFR conditions can be too close to induce different impacts on blood flow. Alternatively, $\dot{V}O_{2}\tau p$ can be increased only to a ceiling point, after which there is no pressure dependence. Nevertheless, this observation suggests that BFR may slow the oxidative metabolism in the early phase of exercise transition in the heavy intensity domain. This is in agreement with previous reports that observed a slower $\dot{V}O_{2}\tau p$ during supine compared with upright leg extension exercise in the moderate intensity domain (MacDonald et al., 1998). However, cycling $\dot{V}O_{2}$ kinetics has previously been shown to be unaffected when comparing upright and supine exercises in a heavy intensity domain similar to the present study (Egaña et al., 2010). Finally, while total $\dot{V}O_{2}$ amplitude significantly increased under BFR, $\dot{V}O_{2}Ap$ remained unaffected. This is in accordance with the aforementioned study that applied BFR in the moderate intensity domain (Knight et al., 2004). Thus, the results of the present work indicate that 40% and 50% of total femoral artery occlusion, applied against a resting measure, can slow the oxidative metabolism during cycling exercise performed in the heavy domain.

Regarding $\dot{V}O_{2}$ SC, the second hypothesis suggested an increase of the $\dot{V}O_{2}Asc^{\prime }$ as a function of BFR pressure. This has been confirmed by the results of the present study indicating significant differences between the conditions. This is in agreement with the majority of works examining the effects of blood flow alteration on the $\dot{V}O_{2}$ kinetics during exercise transitions (Koga et al., 1999; Knight et al., 2004). It has been well-documented that $\dot{V}O_{2}$ SC, which represents an *O*_2_ overconsumption, was related to a deterioration of muscular efficiency in relation to the fatigue process (Henson et al., 1989; Krustrup et al., 2004; Jones et al., 2011). Therefore, the increase of $\dot{V}O_{2}Asc^{\prime }$ during BFR could have been provoked by an exacerbation of fatigue processes. It has been established that BFR produces greater metabolite accumulation in the working muscles, inducing exacerbation of fatigue and elevated muscle activation with type II fibre recruitment (Fatela et al., 2016). The results of the present study can also be partially explained by an increase in anaerobic energy expenditure caused by the application of BFR. This has been suggested by other studies that observed an increase in several anaerobic markers, such as lactatemia during occlusion (Yanagisawa and Sanomura, 2017; Thomas et al., 2018). Accordingly, recent studies have strongly suggested that $\dot{V}O_{2}$ SC is related to the intrinsic metabolic properties of different muscle fibres during heavy exercises (Conde Alonso et al., 2020; Korzeniewski and Rossiter, 2021). Thus, BFR induced a slowdown in the $\dot{V}O_{2}$ primary phase, suggesting a lack tissue oxygenation during the beginning of a cycling exercise transition in the heavy intensity domain and a greater solicitation of anaerobic metabolism to fill the *O*_2_ debt.

Importantly, it has been suggested that the velocity of the primary phase of the *O*_2_ extraction and the blood flow kinetics were inversely related with regard to the Fick principle (Barbosa et al., 2010; Jones et al., 2012). Therefore, the last hypothesis proposed that, to compensate for the alteration of the *O*_2_ delivery kinetics, the application of BFR would cause an acceleration of the *O*_2_ extraction kinetics. The results showed a significant decrease of *TSIMRT* with BFR mostly due to a shorter *TSITD*1, indicating an acceleration of the early extraction process. Concerning *TSI*τ1, there was no significant effect of the BFR pressure. This is consistent with a study that highlighted an increased TD under hyperoxia but no differences regarding τ (Vanhatalo et al., 2010). It has been shown that the amplitude of the primary phase of extraction kinetics was increased during hypoxic conditions to compensate for the reduction in *O*_2_ delivery (Bowen et al., 2013). However, the present study did not indicate significant differences of *TSIA*1′ or *TSIA*1*ss*′ between the conditions.

Furthermore, the results of the present study showed an increase of *TSIA3'* with BFR. Since TSI reflects the dynamic balance between *O*_2_ delivery and utilisation, its progressive reduction when $\dot{V}O_{2}$ SC occurs could be due to an alteration in blood flow as $\dot{V}O_{2}$ continues to increase. Another explanation could be related to an enhanced Bohr effect, allowing greater *O*_2_ diffusion caused by the shift in the *O*_2_ Hb dissociation curve to the right due to the pH reduction at the microvascular level in the muscle (Belardinelli et al., 1995). Moreover, BFR accelerates the early process of the primary phase, as indicated by a shorter MRT, and induces changes in the amplitude of the TSIA*tot* during cycling exercise in the heavy-intensity domain. This suggests an earlier imbalance between *O*_2_ delivery and utilisation during occlusion. The greater effects of BFR occurred during the third phase of the TSI kinetics, probably leading to an increase of the amplitude of $\dot{V}O_{2}$ SC and a greater *O*_2_ extraction related to a higher *O*_2_ utilisation compared to its delivery.

With the assumption that both the TSI and the $\dot{V}O_{2}$ measurements are the products of the working muscles, the kinetics analysis of these two parameters could give valuable information on the dynamic regulation of oxidative metabolism during exercise transitions. The results showed a slowdown of the $\dot{V}O_{2}\tau p$ without a significant change of the *TSI*τ1 during BFR. According to the Fick principle, this observation could be explained by a slower blood flow adaptation when BFR was applied (Grassi and Quaresima, 2016). Some authors observed that, under normal conditions, exercise at higher intensity may tend to slow blood flow and accelerate the *O*_2_ extraction kinetics without slowing $\dot{V}O_{2}\tau p$ (Jones et al., 2012). Their conclusion seems indicate that the slowdown in blood flow was not of enough magnitude to slow the $\dot{V}O_{2}$ kinetics and was compensated by an acceleration of the *O*_2_ extraction. This indicates that the so called “tipping point” mentioned in some other studies was not crossed (Poole et al., 2008; Murias et al., 2014). Regarding the results, it can be speculated that BFR applied during cycling exercise in the heavy-intensity domain shifted the working muscles beyond that “tipping point.” The acceleration of the extraction kinetics could have reached a plateau, hence not permitting compensation for the slowdown in blood flow kinetics, leading to slower $\dot{V}O_{2}$ kinetics.

## Limitations

TSI is sensitive to microvascular blood flow variations induced by thermoregulation (Grassi and Quaresima, 2016). Thus, it is conceivable that this latter phenomenon could have influenced the TSI kinetics measurements. The TSI measurement zone comprised only a small superficial volume of the vastus lateralis muscle. To be confronted with the $\dot{V}O_{2}$, the local TSI was considered to reflect the whole working muscles. However, supporting this extrapolation, similar HHb kinetics were observed between different quadriceps muscles (vastus medialis vs. vastus lateralis muscles) (duManoir et al., 2010). In addition, it was suggested that the relation between *O*_2_ delivery and utilisation is homogenous between these muscles (duManoir et al., 2010). Another limitation of this study is the inability to extrapolate the results to lower-intensity exercise domains. It is important to note given the vast majority of BFR literature uses an applied occlusion during exercise in the low-intensity domain. Finally, this study did not include female healthy trained athletes because several responses such as cardiovascular adaptations differ between males and female (Patel et al., 2021) and may introduce variability. However, extrapolating from these data presents some limits and further studies are needed to compare outcomes, especially because females are underrepresented in this literature.

## Conclusions

This study highlighted that, during the primary phase of the exercise transition, a slowdown of $\dot{V}O_{2}$ kinetics is induced by BFR. A slower $\dot{V}O_{2}$ response can be explained by an insufficient *O*_2_ delivery to the working muscles. Moreover, data seem indicate that working muscles were in an *O*_2_ dependent situation under BFR. The increase of the $\dot{V}O_{2}$ elicited by the $\dot{V}O_{2}$ SC had a dose dependent relationship with the BFR pressure, suggesting a greater oxidative energy cost. This result can be explained by an alteration of muscle efficiency due to the exacerbation of fatigue and accumulation of metabolites. The kinetics of O_2_ extraction also demonstrated an increase in the phase 3 amplitude, indicating enhanced *O*_2_ consumption compared to delivery. Further studies are needed to confirm changes in metabolism and metabolite accumulation with cellular biomarkers regarding emergence of $\dot{V}O_{2}$ SC during BFR. Finally, as a future direction, cellular biomarkers measurements would be helpful to improve our knowledge on the mechanistic determination of the cause of the slowed $\dot{V}O_{2}$, as well as tissue extraction modulations.

## Data Availability Statement

The original contributions presented in the study are included in the article/supplementary material, further inquiries can be directed to the corresponding author.

## Ethics Statement

The studies involving human participants were reviewed and approved by Commission cantonale d'éthique de la recherche sur l'être humain, Canton de Vaud, CER-VD (VD-2017-02193). The patients/participants provided their written informed consent to participate in this study.

## Author Contributions

KS performed the experiments. KS, AS, and FB analysed the data or wrote the manuscript and read and approved the final version of the manuscript. All authors contributed to the article and approved the submitted version.

## Conflict of Interest

The authors declare that the research was conducted in the absence of any commercial or financial relationships that could be construed as a potential conflict of interest.

## Publisher's Note

All claims expressed in this article are solely those of the authors and do not necessarily represent those of their affiliated organizations, or those of the publisher, the editors and the reviewers. Any product that may be evaluated in this article, or claim that may be made by its manufacturer, is not guaranteed or endorsed by the publisher.

## Abbreviations

BFR: blood flow restriction
BFR40: 40% occlusion of the total femoral artery pressure
BFR50: 50% occlusion of the total femoral artery pressure
CON, control (i.e.: exercise conducted without blood flow restriction)
Hb: haemoglobin
HHb: deoxyhaemoglobin
NIRS: near infrared spectrometry
OXPHOS: mitochondrial oxidative phosphorylation system
*O*_2_Hb: oxyhaemoglobin
Pi: inorganic phosphate
TSI: Tissue saturation index
TSI*A*1′: amplitude of phase 1 (TSI kinetics)
TSI*A*2′: amplitude of phase 2 (TSI kinetics)
TSI*A*1*ss*′: amplitude of steady state (TSI kinetics)
TSI*A*3′: amplitude of phase 3 (TSI kinetics)
TSIA*tot*: amplitude of total response (TSI kinetics)
TSIMRT: mean response time of phase 1 (TSI kinetics)
TSI*TD*1: time delay of phase 1 (TSI kinetics)
TSI*TD*2: time delay of phase 2 (TSI kinetics)
TSI*TD*3: time delay of phase 3 (TSI kinetics)
TSIτ1: time constant of phase 1 (TSI kinetics)
$\dot{V}E$: ventilation
$\dot{V}O_{2}$: pulmonary oxygen uptake
$\dot{V}O_{2}Ap$: amplitude of the primary phase of $\dot{V}O_{2}$ kinetics
$\dot{V}O_{2}Asc$: amplitude of the $\dot{V}O_{2}$ slow component
$\dot{V}O_{2}Atot$: amplitude of the total $\dot{V}O_{2}$ response
$\dot{V}O_{2BL},\;\dot{V}O_{2}$: averaged during the last 30 s of the empty pedalling phase
$\dot{V}O_{2}$MRT: mean response time of the primary phase of $\dot{V}O_{2}$ kinetics
$\dot{V}O_{2}$peak: peak oxygen consumption
$\dot{V}O_{2}$SC: $\dot{V}O_{2}$ slow component
$\dot{V}O_{2}TDp$: time delay of the primary phase of $\dot{V}O_{2}$ kinetics
$\dot{V}O_{2}TDsc$: time delay of the $\dot{V}O_{2}$ slow component
$\dot{V}O_{2}\tau p$: time constant of primary phase of $\dot{V}O_{2}$ kinetics
$\dot{V}O_{2}\tau sc$: time constant of slow component of $\dot{V}O_{2}$ kinetics
VT: ventilatory threshold
PΔ10: power equivalent at 10% of the difference between the power at VT1 and the maximal aerobic power.
